# Effects of Phytosterol Ester Supplementation on Egg Characteristics, Eggshell Ultrastructure, Antioxidant Capacity, Liver Function and Hepatic Metabolites of Laying Hens during Peak Laying Period

**DOI:** 10.3390/antiox13040458

**Published:** 2024-04-12

**Authors:** Wenzi Wu, Xin Ma, Rui Chen, Jinghui Fan, Wenxin Ye, Zhuo Chen, Qixin Huang, Lichun Qian

**Affiliations:** 1 Key Laboratory of Animal Nutrition and Feed Science in East China, Ministry of Agriculture, College of Animal Sciences, Zhejiang University, Hangzhou 310058, China; 22217073@zju.edu.cn (W.W.); 22317062@zju.edu.cn (X.M.); 22217014@zju.edu.cn (Q.H.); 2 Hainan Institute of Zhejiang University, Sanya 572025, China; 22317075@zju.edu.cn (R.C.); 22117080@zju.edu.cn (W.Y.); 22217071@zju.edu.cn (Z.C.); 3 Hangzhou Academy of Agricultural Sciences, Hangzhou 310004, China; zigugu@hz.com

**Keywords:** phytosterol esters, egg characteristics, eggshell ultrastructure, antioxidant capacity, liver function, hepatic metabolomics

## Abstract

The aim of this experiment was to investigate the effects of dietary Phytosterol Ester (PSE) supplementation on egg characteristics, eggshell ultrastructure, antioxidant capacity, liver function, hepatic metabolites, and its mechanism of action in Hy-Line Brown laying hens during peak laying period. A total of 256 healthy Hy-Line Brown laying hens were randomly allocated into four groups. The hens in the control group were fed a basal diet, while those in the experimental groups were fed a basal diet further supplemented with 10, 20, and 40 mg/kg PSE, respectively. It was found that the addition of 20 mg/kg and 40 mg/kg PSE to the diets increased egg weight, but decreased egg breaking strength (*p* < 0.05). The addition of PSEs to the diets increased albumen height and Haugh unit in all experimental groups (*p* < 0.05). Electron microscopic observation revealed that the mammillary thickness increased significantly at doses of 20 and 40 mg/kg, but the total thickness decreased, and the effective thickness also thinned (*p* < 0.05). The mammillary width narrowed in all experimental groups (*p* < 0.001). Dietary supplementation with 40 mg/kg PSE significantly increased egg yolk Phenylalanine, Leucine, and Isoleucine levels (*p* < 0.05). In untargeted liver metabolomic analyses, L-Phenylalanine increased significantly in all experimental groups. Leucyl-Lysine, Glutamyl-Leucyl-Arginine, and L-Tryptophan increased significantly at doses of 10 and 20 mg/kg (*p* < 0.05), and L-Tyrosine increased significantly at doses of 10 and 40 mg/kg (*p* = 0.033). Aspartyl-Isoleucine also increased significantly at a dose of 10 mg/kg (*p* = 0.044). The concentration of total protein in the liver was significantly higher at doses of 20 and 40 mg/kg than that of the control group, and the concentrations of total cholesterol and low-density lipoprotein cholesterol were significantly reduced (*p* < 0.05). The concentration of triglyceride and alkaline phosphatase were significantly reduced in all experimental groups (*p* < 0.05). Steatosis and hemorrhage in the liver were also improved by observing the H&E-stained sections of the liver. Concerning the antioxidant capacity in the liver, malondialdehyde concentration was significantly reduced (*p* < 0.05) at a dose of 40 mg/kg. In the ovary, malondialdehyde and nitric oxide concentrations were significantly reduced (*p* < 0.001). In all the experimental groups, plasma nitric oxide concentration was significantly decreased while superoxide dismutase was significantly increased, and total antioxidant capacity concentration was significantly increased (*p* < 0.05) in the 10 mg/kg and 40 mg/kg doses. Metabolomics analyses revealed that PSEs play a role in promoting protein synthesis by promoting Aminoacyl-tRNA biosynthesis and amino acid metabolism, among other pathways. This study showed that the dietary addition of PSEs improved egg characteristics, antioxidant capacity, liver function, and symptoms of fatty liver hemorrhagic syndrome in Hy-Line Brown laying hens at peak laying stage. The changes in liver metabolism suggest that the mechanism of action may be related to pathways such as Aminoacyl-tRNA biosynthesis and amino acid metabolism. In conclusion, the present study demonstrated that PSEs are safe and effective dietary additives as an alternative to antibiotics.

## 1. Introduction

Avian Fatty Liver Hemorrhagic Syndrome (FLHS) is the most common nutrient metabolic disease in the global egg farming industry, characterized by lipid metabolism disorders, liver rupture, and hepatic hemorrhage [1]. FLHS often occurs in commercially caged, high-yielding, and overfed laying hens [2]. The use of cage systems in laying hen farming has led to an increase in FLHS incidence, which accounts for 40% to 70% of laying hen mortality [3]. FLHS typically results in a sharp decline in egg production, shortened peak laying periods, and is the most common non-infectious cause of death [2,4], which has caused serious economic losses to the poultry industry. At the same time, high reproductive performance is essential for poultry to produce healthy offspring and high-quality meat products. Chickens are stress-prone animals, and this is particularly evident in intensive farming, where they are more sensitive to factors such as environment, temperature, transport, and anesthesia treatments, which can cause significant stress injuries [5,6]. High-age breeding birds experience a decline in reproductive performance during breeding, which can be accelerated by oxidative stress and immune imbalance [7,8]. Antibiotics can be effective against oxidative stress, but their overuse can lead to bacterial resistance. Therefore, the development of alternative dietary additives to antibiotics has become a key strategy for solving poultry reproduction problems.

Phytosterols (PSs) are among the top ten functional nutrients recommended by the International Society for Nutrition (ISN) for the future and represent a new raw material for natural health foods that can “lower blood lipids and prevent atherosclerosis” [9]. Phytosterol esters (PSEs) are the product of an esterification reaction between PSs and fatty acids [9,10,11,12]. Since sterol esters can be converted into sterols and fatty acids in the body, they contain the physiological functions of both PSs and fatty acids [13], such as lowering cholesterol, anti-oxidative stress, anti-cancer, anti-inflammatory, growth promotion, and immune regulation, among others [14,15]. In 1999, the U.S. Food and Drug Administration (FDA) approved the addition of PSs and PSEs to food products, allowing for a “beneficial to health” label [13,16]. Phytosterols (esters) are widely used in the cosmetics industry [17,18], and have shown even greater promise in food health care, pharmaceuticals, agriculture, and animal husbandry. In food health care and pharmaceuticals, they are mainly utilized to reduce cholesterol, lower blood lipids, and protect blood vessels [19], and are clinically used to treat non-alcoholic fatty liver disease [20,21,22], protect gastric mucosa, and fight against digestive tract tumors [23,24], among other applications. In the feed industry, they are mainly used as functional feed additives and can reduce the cholesterol content of meat, eggs, and milk products, promote animal growth, and improve yield [25]. PSEs have been reported to improve the quality of laying eggs [26], but studies on their mechanisms have been less explored.

The aim of this study was to investigate the effects of dietary PSE supplementation on egg characteristics, eggshell ultrastructure, antioxidant capacity, liver function, hepatic metabolites, and its underlying mechanism in Hy-Line Brown laying hens at peak lay.

## 2. Materials and Methods

### 2.1. Animals, Experimental Design, and Diets

The experiment was designed as a unidirectional study. A total of 256 healthy Hy-Line Brown laying hens (25 weeks of age) with similar body weight and egg production rate (above 88%) were randomly assigned into 4 groups with eight replicates having eight hens. Each group has eight cages with eight chickens in each cage. All experiments followed the guidelines for animal care and use, and were approved by the Committee for Animal Research at Zhejiang University (Hangzhou, China) (ZJU20240055).

Laying hens in the control group were fed a basal diet, while those in the experimental group received a basal diet containing 10, 20, or 40 mg/kg PSE, respectively [27]. The diets for both control and experimental groups were produced from the same batches of raw materials under identical processing conditions.

Referring to the “Chicken Feeding Standard NY/T33-2004” and the commercial laying hens feeding manual of Hy-Line International, the main composition of laying hens’ feed was determined, and a corn–soybean meal-type basal diet was designed and formulated into powdered compound feeds for testing. The composition and nutrient level of the basal diet are shown in Table 1.

### 2.2. Management

Laying hens in each group were housed under identical conditions. A 14-day pre-test period was followed by a 56-day formal test period. Experimental laying hens were housed in the same closed laying hen house. The laying hens were housed in three-tier ladder-type rearing cages, with four hens per cage (48 cm × 34 cm × 37 cm). Incandescent lamps were used in the house with a light intensity of 30 Lx. Light duration was 15 h/day from 5:00 to 20:00. Temperature was between 28 and 31 °C and relative humidity between 70 and 80%. Hens were fed manually twice daily, and eggs were collected once daily at a fixed time. Feed intake was 115–120 g/bird/day, determined weekly by combining real-time feed intake, egg-laying rate, air temperature, and other factors.

### 2.3. Determination of Performance Parameters

After the start of the experiment, eggs were collected, marked, and weighed weekly. Eggs with damage, deformity, frosted eggshell surface, and unusual weight (≤43 g or ≥78 g) were unqualified. Feed intake was measured per cage and was calculated for each hen. Laying rate, feed-to-egg ratio (F/G), and average egg weight were calculated.

### 2.4. Determination of Egg Quality

Five days before the end of experiment, 40 eggs (5 randomly selected from each replicate) were randomly collected from each group for determination of egg quality. Egg weight, egg breaking strength, albumen height, yolk color depth, eggshell thickness, and Haugh unit were calculated. Yolk samples were removed and stored at −80 °C for determination of amino acid profile, biochemical indexes, and antioxidant capacity. Meanwhile, after egg characteristic measurements, eggshells were collected and stored for observation and determination of eggshell ultrastructure.

### 2.5. Sample Collection

At 56 days, two birds of moderate weight were randomly selected from each replicate. Blood samples (5 mL) were collected from the brachial vein into a 10 mL anticoagulant-free Vacutainer tube (Greiner BioOne GmbH, Kremsmunster, Austria). After standing for 2 h at 37 °C, the serum was centrifuged at 3000 rpm for 15 min at 4 °C, separated, and stored in a −80 °C freezer for further analysis. Anesthesia was administered to the laying hens. The mixture of SumianxinII and ketamine was prepared in a 1:1 equivocate, and the animals were first anesthetized by intramuscular injection at a dose of 0.25 mL/kg body mass, and then injected again by intramuscular injection at a dose of 0.1 mL/kg body mass when the animals became unresponsive, and then after the effective anesthesia had disappeared, an additional dose of half of the total dose (0.35 mL/kg body mass) was given [28]. A 1 cm^3^ liver was removed and fixed in 4% paraformaldehyde. The remaining liver was placed in sample bag and temporarily stored in a liquid nitrogen tank. The samples were then transferred to a freezer at −80 °C for further testing.

### 2.6. Determination of Amino Acid Analysis in Egg Yolk

To prepare the sample, weigh 100 mg and add 1000 μL of extraction solution (methanol:acetonitrile:water = 2:2:1, *v*/*v*/*v*). Shake and mix well. Sonicate the sample in an ice water bath for 10 min and then snap-freeze it in liquid nitrogen for 1 min. Repeat this process three times. Next, separate the supernatant at 13,000 rpm for 15 min at 4 °C. Blow the supernatant dry with a nitrogen blower and then add 100 μL of acetonitrile:water = 1:1 (*v*/*v*) to redissolve. Shake the mixture for 30 s, sonicate it in an ice water bath for 10 min, centrifuge it at 13,000 rpm for 15 min at 4 °C, remove the supernatant, and prepare it for testing.

The LC/MS detection method was adopted, and the chromatographic conditions were as follows: chromatographic column: ACQUITY UPLC^®^ BEH C18 column (2.1 × 100 mm, 1.7 μm, Waters, Milford, Massachusetts (MA), USA), injection volume: 5 μL, column temperature: 40 °C, mobile phases: A-100% H_2_O, 25 mM CH_3_COONH_4_ + 25 mM NH_4_OH, B-100% ACN. The flow rate was 0.3 mL/min. Mass spectrometry conditions: electrospray ionization (ESI) source. Ion source temperature 600 °C, ion source voltage −4500 V or 5500, air curtain gas 20 psi, atomization gas and auxiliary gas both 60 psi. Scanning was performed by multiple reaction monitoring (MRM).

### 2.7. Observation and Determination of Eggshell Ultrastructure

Six eggshells per group were selected for ultrastructural analysis. A piece of shell measuring 1 cm^2^ was cut from the equatorial region of each egg and the shell membranes were removed by soaking in water. The loosely adhering membranes were then gently peeled. To remove the remaining tightly bound membrane fibers, each sample was soaked in a solution containing 6% sodium hypochlorite, 4.12% sodium chloride, and 0.15% sodium hydroxide overnight [29]. The specimen was then rinsed with water and left to dry at room temperature. After the above preparative treatments, each sample was mounted section-side up on an aluminum column and then coated with gold for 1 min in a GVC-2000 Full automatic magnetron ion (Beijing Zhongke Keyi Co., Beijing, China). These samples were examined using a Hitachi Tabletop Microscope TM-1000 (Hitachi, Tokyo, Japan). The cross-sectional length of the eggshell was measured directly in μm at magnifications of 100× and 200×. The total thickness of each sample was measured as the distance from its outermost surface to the point at which the basal cap inserts into the shell membrane. The mammillary thickness was measured as the distance from the basal cap to the point where the mammillary first fused. The effective thickness is obtained by subtracting these two measurements. The mammillary width is the distance between the fusion points on either side of the mammillary [30,31].

### 2.8. Determination of Lipid Metabolites and Antioxidant Capacity

Each liver and ovary sample was weighed accurately to 0.2 g, and 9 times the weight of the specific dilution solution was added. The samples were homogenized (about 60 s) using a fast homogenizer under an ice bath until no particles were visible in the homogenized solution. The prepared homogenized solution was centrifuged at 3000 rpm for 10 min at 4 °C to remove debris. A portion of the resulting supernatant was retained for analysis, and the rest was immediately stored at −80 °C for further determination.

The levels of total cholesterol (TC), low-density lipoprotein cholesterol (LDL-C), high-density lipoprotein cholesterol (HDL-C), triglyceride (TG), total protein (TP), albumin (ALB), and alkaline phosphatase (AKP) were measured in the supernatant of liver tissues using kits produced by the Nanjing Jiancheng Bioengineering Institute (Nanjing, China).

At the same time, catalase activity (CAT), total antioxidant capacity (T-AOC), superoxide dismutase (SOD) activity, malondialdehyde (MDA), and nitric oxide (NO) of serum, liver, and ovary were measured using kits provided by the Nanjing Jiancheng Bioengineering Institute (Nanjing, China).

### 2.9. Histology

Liver tissues were fixed in 4% paraformaldehyde, embedded in paraffin, sectioned, and stained with hematoxylin and eosin (H&E). Histopathological changes were examined using a light microscope (Olympus, Tokyo, Japan) at 200× and 400× magnification. Images were acquired using Cellsens software (version V4.1.1).

### 2.10. Non-Targeted Profiling of Metabolites in Liver

Non-targeted profiling of metabolites in liver tissues of laying hens was performed at the Majorbio Bio-Pharm Technology Co., Ltd. (Shanghai, China). First, a 50 mg solid sample was added to a 2 mL centrifuge tube along with a 6 mm diameter grinding bead. Then, 400 μL of extraction solution (methanol: water = 4:1 (*v*:*v*)) containing 0.02 mg/mL of internal standard (L-2-chlorophenylalanine) was added. The samples were ground using the Wonbio-96c (Shanghai Wanbo Biotechnology Co., Shanghai, China) frozen tissue grinder for 6 min at −10 °C and 50 Hz, followed by low-temperature ultrasonic extraction for 30 min at 5 °C and 40 kHz. The samples were stored at a temperature of −20 °C for 30 min, then centrifuged for 15 min at 13,000× *g* and 4 °C [32]. The resulting supernatant was then transferred to the injection vial for LC-MS/MS analysis.

The sample was analyzed using LC-MS/MS on a Thermo UHPLC-Q Exactive HF-X system equipped with an ACQUITY HSS T3 column (100 mm × 2.1 mm i.d., 1.8 μm; Waters, Milford, MA, USA) at Majorbio Bio-Pharm Technology Co., Ltd. (Shanghai, China). The sample was separated on the column and passed into mass spectrometry using 3 μL. Mobile phase A consisted of 95% water and 5% acetonitrile (with 0.1% formic acid), while mobile phase B consisted of 47.5% acetonitrile, 47.5% isopropanol, and 5% water (with 0.1% formic acid). The flow rate was 0.40 mL/min, and the column temperature was 40 °C [33].

The Thermo UHPLC-Q Exactive HF-X Mass Spectrometer (Thermo Fisher Scientific, Massachusetts, MA, USA) was used to collect the mass spectrometric data. The electrospray ionization (ESI) source was operated in both positive and negative modes. The mass scan range was between 70 and 1050 *m*/*z*. The sheath gas flow rate was set to 50 psi, while the auxiliary gas flow rate was set to 13 psi. The auxiliary gas heating temperature was set to 425 °C. The positive-mode ion-spray voltage floating (ISVF) was set to 3500 V, and the negative-mode ISVF was set to −3500 V. The temperature of the ion transfer tube was maintained at 325 °C, and the normalized collision energy was varied between 20, 40, and 60 V in a cyclic manner. The primary mass spectrum had a resolution of 60,000, while the secondary mass spectrum had a resolution of 7500 [34]. The data were collected in Data Dependent Acquisition (DDA) mode.

After the onboarding was completed, the LC-MS raw data were imported into the metabolomics processing software Progenesis QI (Version 3.0, Waters Corporation, Milford, CT, USA) for baseline filtering, peak identification, integration, retention time correction, and peak alignment, and finally, a data matrix of retention time, mass-to-charge ratio, and peak intensity was obtained. The metabolites were identified by searching databases, and the main databases were the HMDB (http://www.hmdb.ca/ (accessed on 20 June 2023)), Metlin (https://metlin.scripps.edu/ (accessed on 20 June 2023)), and Majorbio Database. The data matrix obtained by searching the database was uploaded to the Majorbio cloud platform (https://cloud.majorbio.com (accessed on 20 June 2023)) for data analysis.

Principal Component Analysis (PCA) and Orthogonal Least Squares Discriminant Analysis (OPLS-DA) were performed on the preprocessed data matrices using the ropls package in R (Version 1.6.2). The significantly different metabolites were selected based on the variable weight values (VIP) obtained from the OPLS-DA model and the student’s *t*-test *p*-value. Metabolites with VIP > 1 and *p* < 0.05 were considered significant. Metabolic pathways associated with differential metabolites were identified through metabolic pathway annotation using the KEGG database (https://www.kegg.jp/kegg/pathway.html (accessed on 20 June 2023)). Pathway enrichment analyses were conducted using the Python package scipy.stats [35].

### 2.11. Statistical Analysis

The data were obtained through at least three repetitions of the experiment. The statistical analyses were conducted using IBM SPSS Statistics 25.0 (SPSS Inc., Chicago, IL, USA). The data were presented as means with standard error of the mean (SEM). Statistical differences between two or more groups were determined using One-way ANOVA and LSD test (*p* < 0.05). The correlation between the variables was analyzed using bivariate Pearson correlation coefficients.

## 3. Results

### 3.1. Performance Parameters

Table 2 shows that PSE dietary supplementation improved the performance parameters of laying hens at peak laying. Prior to the trial, all groups had similar feed intake, laying rate, feed-to-egg ratio, and average egg weight (*p* > 0.05). At the end of the experiment, 20 mg/kg and 40 mg/kg PSE significantly (*p* < 0.001) increased mean average egg weight compared with the control group, by 3.82% and 4.12%, respectively. No differences were observed in other performance parameters (laying rate, feed intake, and feed-to-egg ratio) among treatments (*p* > 0.05).

### 3.2. Egg Quality

As shown in Table 3, PSE supplementation improved albumen height, and Haugh unit (*p* < 0.05) but reduced egg-breaking strength. No differences were observed in other egg quality parameters (yolk color depth and eggshell thickness) among treatments (*p* > 0.05).

### 3.3. Ultrastructure of Eggshell

**Figure 1:**
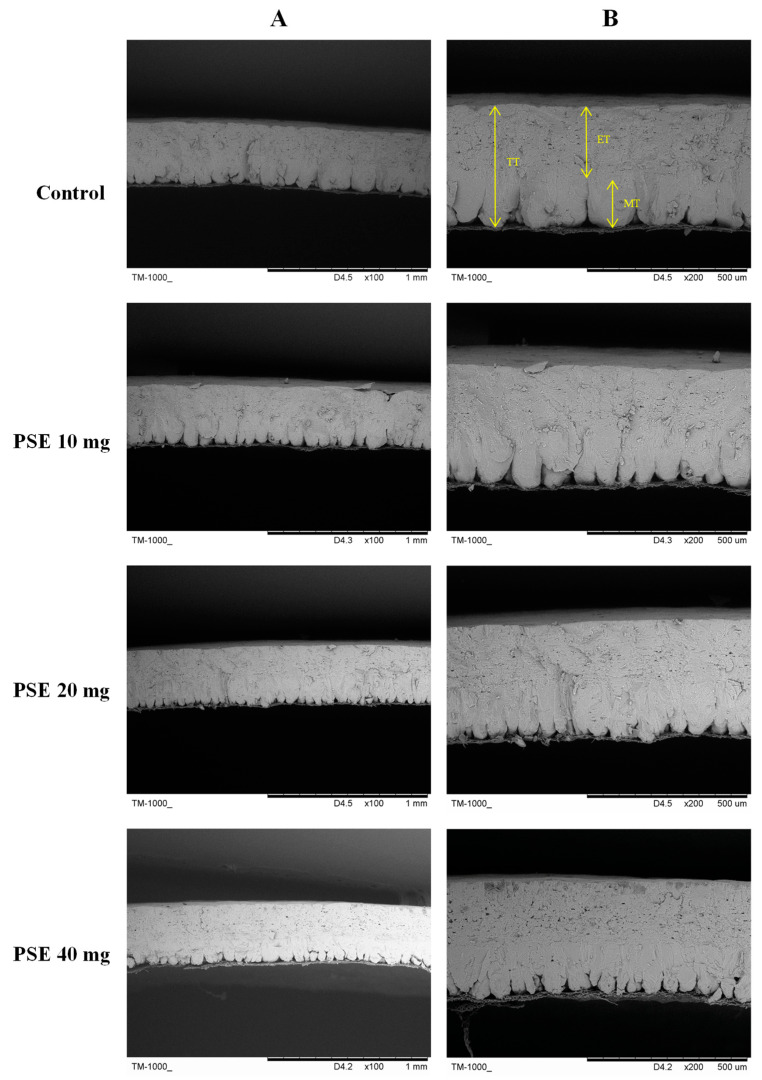
Ultrastructure of eggshell cross-section: (**A**) magnification 100× and (**B**) magnification 200×. TT: Total thickness; ET: Effective thickness; MT: Mammillary thickness.

Measurements were repeated three times for each eggshell sample (*n* = 6) and averaged for statistical analysis. As shown in Figure 1, the effective layer of eggshells in the control group was structurally dense, with wider and shorter mammillary, while the eggshells of the experimental group were more sparsely structured, with narrower and taller mammillary, compared with those of the control group.

As indicated in Table 4, the mammillary thickness increased significantly at doses of 20 and 40 mg/kg, but the total thickness decreased, and the effective thickness also thinned (*p* < 0.05). The mammillary width narrowed in all experimental groups (*p* < 0.001). Representative images of the eggshell ultrastructure are shown in Figure 1.

### 3.4. Amino Acid Profile of Egg Yolk

Differences in concentrations of some amino acids in egg yolk were observed as an effect of PSE dietary supplementation (Table 5). The addition of 40 mg/kg PSE significantly increased Isoleucine, Leucine, and Phenylalanine contents in egg yolk compared to the control (*p* < 0.05), but had no significant effect on contents of any other essential amino acids (Valine, Glycine, Alanine, Serine, Proline, Threonine, Asparagine, Aspartic, Homocysteine, Glutamine, Lysine, Glutamic, Methionine, Histidine, Arginine, Tyrosine, and Tryptophan) among treatments (*p* > 0.05).

As indicated in Table 6, significant differences among groups were also observed in untargeted metabolomic analyses of the liver for some amino acids. L-Phenylalanine increased significantly in all experimental groups. Leucyl-Lysine, Glutamyl-Leucyl-Arginine, and L-Tryptophan increased significantly at doses of 10 and 20 mg/kg (*p* < 0.05), and L-Tyrosine increased significantly at doses of 10 and 40 mg/kg (*p* = 0.033). Aspartyl-Isoleucine also increased significantly at a dose of 10 mg/kg (*p* = 0.044).

### 3.5. Phytosterol Esters Improved Fatty Liver Hemorrhagic Syndrome in Laying Hens

The effects of PSE dietary supplementation on liver biochemical parameters are shown in Table 7. Compared with the control group, PSEs at dosages of 20 and 40 mg/kg increased the concentration of serum TP (*p* = 0.002), and decreased the AKP, TC, LDL-C, and TG concentrations (*p* < 0.05). No significant differences in ALB were found between the experimental group and the control group (*p* > 0.05). HDL-C tended to increase but did not reach a significant level.

**Figure 2:**
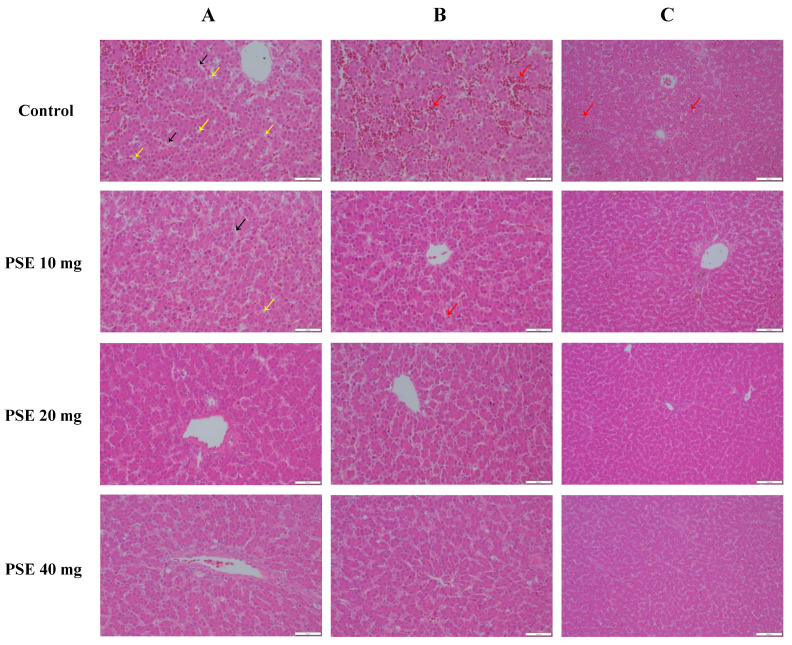
Representative images of H&E staining of liver sections: (**A**) fatty deposition, magnification 400×, Scale bar = 50 μm. The yellow arrows point to fat particles, and the black arrows point to the chaotic array of hepatocytes and hepatic sinusoidal; (**B**) hepatic stasis, magnification 400×, Scale bar = 50 μm; (**C**) hepatic stasis, magnification 200×, Scale bar = 100 μm; The red arrow points to stasis in the liver tissue.

Examining the H&E staining of liver sections, all groups exhibited some degree of hepatic lesions. The control group showed the most severe pathology, with abundant round vacuoles (i.e., fat particles, shown by yellow arrows) in hepatocytes and ill-defined, chaotically arranged hepatocytes and hepatic sinusoidal, as shown by black arrows (Figure 2A). In contrast, only small numbers of round vacuoles were observed in the PSE 10 mg group, while the PSE 20 mg and PSE 40 mg groups had significantly fewer fat particles compared to the control. Hepatocytes and hepatic sinusoidal in these groups also appeared tidier and more organized. Hepatocytes were fuller with regular morphology and clear nuclei.

The H&E-stained sections showed large numbers of stasis in control liver tissue (Figure 2B,C, shown by red arrows). Comparatively, stasis was significantly reduced in laying hens receiving phytosterol esters, even more so at 20 mg/kg and 40 mg/kg additions.

These results suggest that PSEs can effectively attenuate hepatocellular steatosis and alleviate hepatic stasis, thereby ameliorating fatty liver hemorrhagic syndrome.

### 3.6. Antioxidant Capacity

Table 8 shows the MDA, NO concentration, and antioxidant enzyme activity in the liver, ovary, and plasma of laying hens at peak lay (above 88%). In the liver, the addition of 40 mg/kg PSE significantly decreased MDA concentration (*p* < 0.05). There was no significant difference among all groups for liver T-AOC, CAT, SOD, and NO concentration (*p* > 0.05).

In the ovary, MDA decreased significantly with increasing PSEs from 0 to 40 mg/kg (*p* < 0.001). Compared to 0 mg/kg, NO activity significantly decreased with 10–40 mg/kg PSE (*p* < 0.001). However, T-AOC concentration was not affected by treatments (*p* > 0.05).

In plasma, SOD concentrations were significantly increased, and NO concentrations were significantly decreased (*p* < 0.05) in all experimental groups. Dietary supplementation with 10 and 40 mg/kg PSE significantly (*p* < 0.05) increased T-AOC concentrations. There was no significant difference in plasma MDA among groups (*p* > 0.05).

### 3.7. Phytosterol Esters Alter Liver Metabolites in Laying Hens

#### 3.7.1. Comparative Analysis of Samples

**Figure 3:**
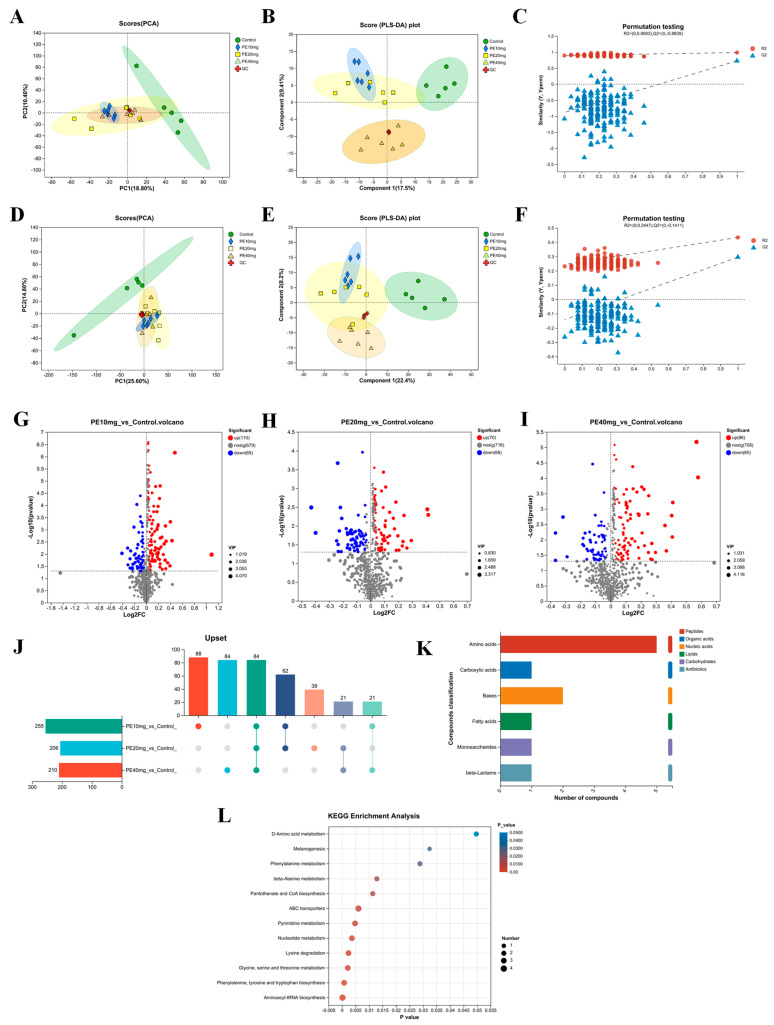
Phytosterol esters alter the metabolome of the liver of laying hens: (**A**–**C**) positive-ion mode: PCA analysis, PLS-DA analysis, PLS-DA substitution test; (**D**–**F**) negative-ion mode: PCA analysis, PLS-DA analysis, PLS-DA substitution test; (**G**–**I**) Differential metabolite volcano plots; (**J**) metabolite Upset plots: The bar chart in the lower left corner of the figure is a statistic of the number of its own elements for each metabolic set. The bar chart on the right is the statistical result of the number of elements after the intersection of various metabolic sets, the single point at the bottom represents the element unique to a certain metabolic set, and the lines between the points represent the intersection unique to the metabolic set; (**K**) classification of KEGG compounds; (**L**) Bubble plots showing KEGG enrichment analysis.

To investigate liver metabolome functional changes, liver samples were analyzed using LC-MS. Principal component analysis (PCA) showed 10.40% and 18.80% variances by PC1 and PC2, respectively, with a significant separation between the control and three experimental groups in score plots (Figure 3A,D). Partial least squares discriminant analysis (PLS-DA) further analyzed metabolic profile changes for each treatment group, showing greater separation of samples between groups indicating more significant classification effects (Figure 3B,E). The PLS-DA score plot showed 9.41% and 17.5% variances by components 1 and 2, respectively, with better differentiation ability than PCA. The control and three experimental groups samples showed more significant group separation on the PLS-DA score plots, with a slight overlap between the experimental groups, indicating PSEs have a regulatory effect on liver metabolism in laying hens during peak production. As shown in Figure 3C,F, decreasing replacement retention led to reduced R2 and Q2, and an upward trend in the regression line, indicating successful model replacement and no overfitting.

#### 3.7.2. Analysis of Differential Metabolites

To further elucidate PSEs’ effects on metabolites, differential metabolites were screened using two-comparison analyses with the statistical method, visualized via volcano plots. Based on the *t*-test (*p* < 0.05), S-plots (Vip > 1.0), and fold change (FC) ≥ 1, LC-MS-derived metabolites were identified. A total of 284 differential metabolites were obtained from 1172 total metabolites, including PE 10 mg_vs_Control, PE 20 mg_vs_Control, and PE 40 mg_vs_Control sets.

Figure 3G shows the visualized volcano plot of differential metabolites between the experimental and control groups with dietary addition of 10 mg/kg PSE (PE 10 mg_vs_Control metabolite set). In Figure 3G, red dots indicate 110 up-regulated metabolites, blue dots indicate 65 down-regulated metabolites, and gray dots indicate 679 metabolites with no significant differences.

Figure 3H shows the visualized volcano plot of metabolites that differed between the experimental and control groups with dietary addition of 20 mg/kg PSE (PE 20 mg_vs_Control metabolite set). In Figure 3H, red dots indicate 70 up-regulated metabolites, blue dots indicate 68 down-regulated metabolites, and gray dots indicate 716 metabolites with no significant differences.

Figure 3I shows a visualized volcano plot of the metabolites that differed between the experimental and control groups with dietary addition of 40 mg/kg PSE (PE 40 mg_vs_Control metabolite set). In Figure 3I, red dots indicate 86 up-regulated metabolites, blue dots indicate 60 down-regulated metabolites, and gray dots indicate 708 metabolites that were not significantly different.

#### 3.7.3. Metabolic Set Analysis

Three metabolic sets—PE 10 mg_vs_Control, PE 20 mg_vs_Control, and PE 40 mg_vs_Control—were analyzed via Venn analysis and visualized using UpSet plots (Figure 3J). The plot depicts the number of metabolites in each set and their overlapping relationships. Taking the intersection of the three sets yielded 84 metabolites potentially impacted by PSE addition.

KEGG compound classification was performed on 84 metabolites, and the results are shown in Figure 3K. Among them, five amino acids were identified: L-Lysine, L-Tryptophan, N-Formyl-L-Methionine, L-Phenylalanine, L-Tyrosine; there are two bases belonging to the nucleic acids, Cytosine and Uracil; in addition, Carboxylic acids, fatty acids, monosaccharides, and beta-Lactams are all of one the kind, Muramic acid wall acid, Hickory acid Behenic acid, Isocitrate, and Penicillin G, respectively.

#### 3.7.4. Metabolic Pathway Analysis

To elucidate the mechanism of PSEs’ effects on the organismal metabolism, we subjected 84 differential metabolites to KEGG pathway enrichment analysis, analyzing the metabolic pathways involving the differential metabolites in liver tissue. As shown in Figure 3L, the horizontal coordinate indicates pathway importance, the vertical coordinate is the KEGG pathway, and bubble size represents the number of differential metabolites enriched to the metabolic set in that pathway. A total of 12 pathways were screened using *p* < 0.05 (Table 9). The results show that PSEs primarily affect the metabolic pathway by influencing Aminoacyl-tRNA biosynthesis; Phenylalanine, tyrosine, and tryptophan biosynthesis; Glycine, serine, and threonine metabolism; Lysine degradation; Nucleotide metabolism; ABC transporter metabolism; Pantothenate and CoA biosynthesis; beta-Alanine metabolism, Phenylalanine metabolism; Melanogenesis; D-Amino acid metabolism; and other pathways that fulfill their physiological roles.

### 3.8. Correlation Analysis

The correlations between egg quality, antioxidant capacity, biochemical indexes, and liver metabolites were analyzed to further explore the potential mechanism of PSEs.

As shown in Table 10, egg weight and Haugh unit were negatively correlated with NO in plasma and MDA, and NO concentration in the ovary. Egg-breaking strength was positively correlated with the plasma’s NO and the ovary’s MDA and NO concentrations. Albumen height was negatively correlated with the liver’s MDA, plasma’s NO, and ovary’s MDA and NO concentrations.

Table 11 shows the correlation analysis between egg characteristics and liver biochemical indexes. Egg weight was positively correlated with liver TP content but negatively correlated with TG and LDL contents. Egg breaking strength was positively correlated with liver TC, TG, and LDL contents, but negatively correlated with TP content. Albumen height was positively correlated with liver TP content but negatively correlated with TC, TG, and LDL contents. Haugh unit was positively correlated with liver TP content but negatively correlated with TG content.

**Figure 4:**
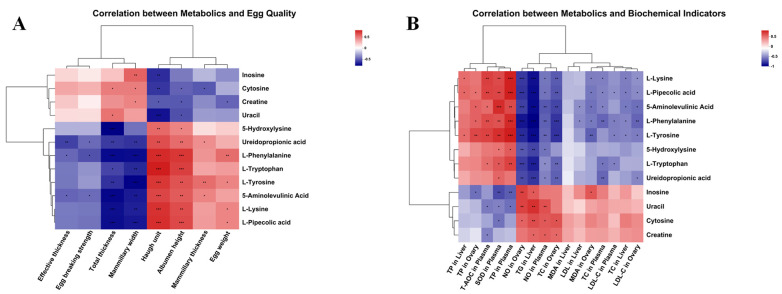
Liver metabolites are linked with egg quality and biochemical indicators: (**A**) Correlation between egg quality and metabolic biomarkers. (**B**) Correlation between biochemical indicators and metabolic biomarkers. In the heatmap of the correlation coefficient, the red represents positive correlations and the blue represents negative correlations (* *p* ≤ 0.05, ** *p* ≤ 0.01, *** *p* ≤ 0.001).

The metabolites of 12 pathways with smaller *p*-values of metabolic pathways were selected in the set of differential metabolisms, and correlation analysis was performed with indicators with significant differences (egg characteristics, eggshell ultrastructure, and biochemical indicators), and the results are shown in Figure 4.

Figure 4A shows a significant negative correlation between total thickness and mammillary width with metabolites 5-Hydroxylysine, Ureidopropionic acid, L-Phenylalanine, L-Tryptophan, L-Tyrosine, 5-Aminolevulinic Acid, L-Lysine, and L-Pipecolic acid, except mammillary width, which is not significantly negatively correlated with 5-Hydroxylysine. Haugh unit and albumen height showed a significant positive correlation with the above metabolites. Total thickness was positively correlated with Cytosine and Uracil, and mammillary width was positively correlated with Inosine, Cytosine, and Creatine. Effective thickness and egg-breaking strength were negatively correlated with 5-Aminolevulinic Acid, Ureidopropionic acid, and L-Phenylalanine. Mammillary thickness was positively correlated with Ureidopropionic acid, L-Tyrosine, and 5-Aminolevulinic Acid and negatively correlated with Cytosine. Egg weight was positively correlated with L-Phenylalanine, L-Tyrosine, L-Lysine, and L-Pipecolic acid and negatively correlated with Creatine.

As evident from Figure 4B, TP in the liver and ovary, and TP, SOD, and T-AOC in Plasma showed a positive correlation with the metabolites 5-Hydroxylysine, Ureidopropionic acid, L-Phenylalanine, L-Tryptophan, L-Tyrosine, 5-Aminolevulinic Acid, L-Lysine, and L-Pipecolic acid, and negative correlation with Inosine, Uracil, Cytosine, and Creatine. Opposite correlations were observed in NO, MDA, TC, and LDL-C in the ovary, TG, MDA, LDL-C, and TC in the liver, and NO, TC, and LDL-C in plasma.

## 4. Discussion

PSs promote protein synthesis and body growth [14]. Animal growth agents containing PSs are resistant to temperature and enzymatic breakdown, making them suitable as feed mixes or additives. PSs reduce phytohormone sensitivity to temperature, increase in vivo resistance to catabolism, and promote protein synthesis, enhancing animal growth [36]. As documented by Zhou et al. [37], PSs bind lipids to form sterol-ribonucleoprotein complexes, stimulating DNA transcription, inducing protein synthesis, and regulating growth. In this study, PSEs were prepared by esterifying phytosterol with lard or palm oil using inorganic salts and organocatalysts [38]. These esterified PSs exhibit significantly higher water and oil solubility while retaining the physiological functions of PSs [13], presenting broad prospects as new additives. The experiment investigated the effects of adding PSEs to the diet on the egg production performance of Hy-Line Brown laying hens during peak laying, and further studied the mechanisms of action.

### 4.1. Phytosterol Esters Improve Egg Characteristics in Laying Hens by Promoting Amino Acid Anabolism

Egg characteristics indicators include eggshell strength, eggshell thickness, albumen height, Haugh unit, and yolk color, with eggshell strength and thickness indicating egg fragility, albumen height reflecting egg white consistency, Haugh unit representing egg freshness, and yolk color indicating yolk pigmentation. Egg appearance, yolk color, and freshness directly affect consumer purchasing decisions, making egg characteristics indicators critical for egg production [39]. This study found dietary PSE supplementation increased egg weight at doses of 20 and 40 mg/kg and increased albumen height and Haugh units, but decreased shell strength in all experimental groups (*p* < 0.05) in Hy-Line Brown laying hens at peak production. Zhang et al. [26] showed PSEs increased egg weight, yolk color, eggshell weight, and surface area in Jingfen No. 6 laying hens at the end of lay, with albumen height and Haugh unit increasing linearly with additive concentration, but not significantly. Qing [13] reported that dietary supplementation with PSEs increased yolk weight and yolk proportion in Japanese quail (*p* < 0.05), while egg breaking strength was slightly decreased and albumen height and Haugh unit were gradually increased, but none reached significance. These findings are in accordance with the results of the present study, suggesting PSEs beneficially enhance egg characteristics with some differences possibly due to different animal breeds/strains. The Hy-Line Brown laying hens used in the present study are high-yielding, bred by Hy-Line International Company in the United States [40], and are one of the more popular egg-laying hen breeds, widely recognized in international farming. Zhou et al. [41,42] showed that 20, 80, 400, and 800 mg/kg PS had no significant effect (*p* > 0.05) on egg production rate, feed intake, egg production, or feed-to-egg ratio of Hy-Line Brown laying hens. Wang et al. [43] observed that the addition of 5, 10, 20, and 40 mg/kg PS to the diets of late-stage laying hens did not significantly affect the average egg quality or egg production rate (*p* > 0.05), but could reduce the mortality rate, with a significant reduction in the experimental group at 20 mg/kg (*p* < 0.05). The results of these two studies differed from those in the present study, possibly due to differences in the number of additives and period of addition. In the present study, PSE was added at a dose of 10–40 mg/kg and was added during the peak laying period. Zhou et al. added 20–800 mg/kg and Wang et al. added it at the late stage of egg production. PSs have been reported to induce no effect on reproduction and growth at high doses, and phytosterol intake can reduce cholesterol in egg yolks [44], but excessive cholesterol addition increases yolk cholesterol [41]. Chang et al. [45] found that high phytosterol doses significantly affected yolk cholesterol, increasing linearly with dose, while serum and liver cholesterol showed no significant change. Wang et al. [43] showed PSs also affect reproductive hormone levels, which can affect egg quality. In summary, we believe that different additive amounts and addition times may affect the effect of phytosterol esters, but the specific mechanism needs to be further investigated.

The above results indicated a promoting effect of PSEs on egg quality, and we further investigated its mechanism. Haugh unit and albumen height are important indicators for evaluating egg characteristics [46]. Haugh unit is an important indicator for evaluating the freshness and protein content of eggs, and a high Haugh unit indicates fresh eggs with high protein content and little nutrient change within the egg. As egg weight, albumen height, and Haugh unit increased in this study, and PSs promote protein synthesis, we speculated a possible connection with yolk amino acid metabolism and further determined the content of 20 essential amino acids in egg yolk. The results showed that the addition of 40 mg/kg PSE to the ration significantly increased Phenylalanine, Leucine, and Isoleucine levels in egg yolks (*p* < 0.05). Coincidentally, in our non-targeted metabolomics analysis of the liver, we found metabolites related to amino acid metabolic pathways, such as L-Phenylalanine, Leucyl-Lysine, Glutamyl-Leucyl-Arginine, Aspartyl-Isoleucine, L-Tryptophan, L-Tyrosine, etc., differed significantly between groups. Yuan’s [39] experiment results showed groups with 1.27% arginine had a higher Haugh unit and albumen height, indicating appropriate arginine level in the ration could improve egg characteristics. Therefore, we hypothesized that PSEs improved egg characteristics by promoting the amino acid synthesis pathway in laying hens.

Eggshell quality is crucial for poultry eggs, not only to protect the intrinsic quality of the eggs, but also to reduce shell breakage and economic losses during transport. The eggshell is a highly ordered structure, from inside out: inner and outer matrix membranes, papillary layer, fenestrated layer, vertical crystal layer, and the gelatinous protective membrane, the latter three forming the effective layer [47,48]. the eggshell ultrastructure determines mechanical properties and quality [49,50]. To understand decreased eggshell strength in this study, we used electron microscopy to observe the ultrastructure. The results showed the experimental group significantly increased mammillary thickness (*p* < 0.05), but total thickness decreased (*p* < 0.05), effective thickness thinned (*p* < 0.05), and mammillary narrowed (*p* < 0.001). A review of the literature [51] showed that some researchers observed that eggshell formation is closely related to calcium flow in laying hens via electron microscopy. The more calcium deposited, the more pronounced the eggshell stratification and the stronger the structure, suggesting that eggshell calcium deposition definitely had a positive effect on the formation of ultrastructure [52]. The addition of PSEs to laying hens’ feed may impact calcium absorption. Ma et al. [53] found soy stanols inhibited prostatic interstitial contraction via calcium ion inward flow inhibition. PSEs and cholesterol share similar chemical structures, competing for absorption in the intestine, indirectly affecting calcium absorption. Zhao [54] found cholesterol inhibited IP3Rs-mediated calcium transport to mitochondria, in turn, inhibiting mitochondrial function, and ultimately suppressing hepatocellular carcinoma proliferation and metastasis. Wang [55] demonstrated cholesterol significantly inhibits the calcium-activated chloride channel of TMEM16A in HEK293 cells, binding to TMEM16A via the K588 site on the fifth transmembrane region, matching common cholesterol-binding protein motifs and likely the cholesterol-binding site on TMEM16A’s calcium-activated chloride channel. These studies collectively show links between PSEs, eggshell ultrastructure, and Ca ions, warranting further mechanistic investigation. Therefore, to optimally utilize PSEs, they should be administered in proper proportion and dosage, or calcium preparations added to feed at appropriate levels with correct nutritional ratios to maintain laying hen health and nutrient balance.

### 4.2. Phytosterol Esters Effectively Reduced Fatty Liver in Laying Hens

The liver is the primary organ of lipid metabolism and serves as the main site of cholesterol and triglyceride synthesis. It secretes and regulates a variety of active molecules and completes fatty acid oxidation and cholesterol degradation. In recent years, researchers have confirmed through various experiments that PSEs have cholesterol-lowering mechanisms [56]. Firstly, PSE inhibits intestinal cholesterol absorption. PSE and cholesterol have similar structures but different side chain groups. When both exist in the intestine, PSE competitively inhibits cholesterol absorption. Secondly, PSE affects cholesterol transport. Cholesterol in the blood is transported by low-density lipoprotein (LDL). PSE affects LDL formation, thereby affecting cholesterol transport. Thirdly, PSE affects cholesterol synthesis and secretion. Experimental studies have demonstrated that PSE reduces the activity of enzyme systems related to cholesterol synthesis [57]. In this study, adding PSEs to the feed significantly increased the liver TP concentration and significantly decreased the concentrations of TC, LDL-C, TG, and AKP (*p* < 0.05). AKP is an important biochemical indicator of liver function and reflects the degree of liver damage [58]. There was a tendency for ALB and HDL-C to decrease in the experimental group compared to the control group, but it was not significant (*p* > 0.05).

Plasma total cholesterol (TC) is the sum of cholesterol contained in all lipoproteins in the plasma. TC can be divided into HDL-C and LDL-C, and the reduction in LDL-C by PS and PSE is divided into five periods, i.e., the early period, the mixed period, the cellular period, the transport period, and the new discovery period; however, the underlying mechanism is still unclear and needs to be further investigated [59]. Qing [13] showed that the plasma cholesterol concentration of quail decreased significantly with the increase in PSE addition. The results of previous studies showed that PSEs reduced intestinal absorption of cholesterol, thereby reducing plasma cholesterol concentration [60]. Ding et al. [27] showed that the addition of 20 and 40 mg/kg of PSs reduced TG, TC, and LDL-C in the blood lipid levels of white feather broilers. Wen et al. [61] showed that the addition of PSs reduced the relative mass of abdominal lipids in 30-week-old Issa Brown laying hens and reduced TG, TC, HDL-C, and LDL-C levels in the serum of 4-week-old chickens. Jia et al. [62] showed that the addition of 40 mg/kg PS to broiler diets reduced blood LDL-C and TC levels and increased serum TP content, improving the antioxidant capacity of the chicken organism. Sun [63] showed that PSs significantly reduced the levels of TC and LDL-C, and significantly increased the levels of SOD and GSH-PX in the plasma. PSs promoted the growth of broiler ducks and lowered the feed-to-meat ratio to a certain extent. The above studies are almost in agreement with our results. We analyzed the relationship between egg weight, albumen height, Haugh unit, and hepatic lipid metabolism by Pearson’s correlation, which showed that egg weight was positively correlated with the TP content in the liver and negatively correlated with the TG and LDL content. Albumen height was positively correlated with TP content in the liver and negatively correlated with TC, TG, and LDL content. Haugh unit was positively correlated with TP content in the liver and negatively correlated with TG content. The results of the studies all indicated that the application of PSs or PSEs in poultry has the efficacy of lowering cholesterol levels, regulating growth, and promoting health, and at the same time, these efficacious feed additions may further enhance the egg-laying performance of laying hens and improve the quality of eggs.

Avian fatty liver hemorrhagic syndrome (FLHS) is the most common non-infectious cause of death in caged laying hens [4] and a pressing problem in the farming industry, with an elevated incidence of FLHS often leading to a sharp decline in egg production in hen flocks [3] and shortened peak egg production. It is characterized by lipid metabolism disorders, liver rupture, and hepatic hemorrhage [1,2]. The laying hens in this study were also caged, so to investigate whether PSEs had an ameliorative effect on FLHS, we performed H&E-stained sections of the livers. The results showed that fat grains were very abundant in the hepatocytes of the control group, and the boundaries between hepatocytes and hepatic blood sinusoids were unclear and disorganized. In the experimental groups, there were a few rounded vacuoles in the 10 mg/kg group and fewer fat grains in the 20 mg/kg and 40 mg/kg groups, and the hepatic sinusoidal were neatly arranged, and the hepatocytes were more full, with regular morphology and clear nuclei. At the same time, a large number of stases were observed in the liver tissue of control group, and the stases of the experimental group were significantly reduced, and the improvement in the group with the addition of 20 mg/kg and 40 mg/kg was more significant. Liu [64] conducted similar experiments on rats, and pathological observation of the liver sections revealed significantly reduced hepatic steatosis in the PSE-added group versus the high-fat model group. Necrosis and inflammation were also diminished. Therefore, they inferred PSEs may mitigate or eliminate the “first blow” of fatty liver formation by lowering blood lipids, and have a restorative effect on high-fat diet-induced hepatocyte damage in rats, reducing steatosis, inflammation, and necrosis in hyperlipidemic rats. These results align with ours, indicating that PSEs can reduce hepatocellular steatosis and hepatic stasis, reducing the incidence of FLHS to some degree. Our study also provides a novel solution to address the most common nutritional metabolic diseases in poultry farming.

### 4.3. Phytosterol Esters Improve Antioxidant Capacity in Laying Hens

The environment contains a wide variety of factors that may cause stress in animals, including external factors such as light, temperature, human actions, breeding density, etc., and internal factors such as diseases and toxins in feed [65]. The rapid growth and high metabolic rate production patterns that accompany modern poultry promote the production of reactive oxygen species (ROS) in laying hens, enhancing oxidative stress [66,67]. Once the rate of ROS production exceeds the binding rate of its own antioxidant system, it leads to lipid oxidation, resulting in the destruction of unsaturated fatty acids in cell membranes, amino acids in proteins, and nucleic acids in DNA. This damages the integrity of cell membranes and cellular molecules, and affects nutrient absorption [68]. Oxidation can accelerate the aging of the organism, leading to impaired organ function, decreased hormone levels, decreased enzyme activity, and other problems. Under stress conditions, laying time is delayed, and the soft egg rate significantly increases in laying hens [69].

In the present study, an addition of 40 mg/kg PSE significantly decreased MDA concentration in the liver (*p* > 0.05), but no significant difference in T-AOC, CAT, SOD, and NO concentration among all groups (*p* > 0.05). In the ovary, MDA decreased significantly with increasing PSEs from 0 to 40 mg/kg (*p* < 0.001). Compared to 0 mg/kg, NO activity significantly decreased with 10–40 mg/kg PSE (*p* < 0.001). However, T-AOC concentration was not affected by treatments (*p* > 0.05). In plasma, SOD concentrations were significantly increased, and NO concentrations were significantly decreased (*p* < 0.05) in all experimental groups. Dietary supplementation with 10 and 40 mg/kg PSE significantly (*p* < 0.05) increased T-AOC concentrations. There was no significant difference in plasma MDA among groups (*p* > 0.05). Zhang’s [26] test results were similar, with increased T-SOD and CAT activities in the liver and plasma and decreased liver MDA content in laying hens fed dietary PSEs. Gong et al. [70] reported that feed PS addition significantly reduced hepatic MDA content and significantly increased hepatic T-SOD and AKP activities, but had no significant effect on CAT activity. Yuan’s [71] study results showed that adding 20 and 40 mg/kg PS to the diet reduced malondialdehyde accumulation and increased glutathione peroxidase (GSH-PX) concentration compared to the control (*p* < 0.05). Huang et al. [72] added 100, 200, and 300 g/t PS to the diet of Qingyuan jatropha chickens and found increased serum SOD and GSH-PX activities, reduced MDA content and enhanced antioxidant capacity. Li et al. [73] found that the addition of 20 or 40 mg/kg PS to tilapia feed increased both SOD and CAT activities in the liver compared to the control, but when added at 160 mg/kg, both activities were reduced compared to the control. It can be inferred that there is a dose relationship between PSs and antioxidant enzyme activities, and the appropriate addition amount has a positive effect on antioxidant capacity. Qing [13] reported that the addition of 150–300 mg/kg PSE to the ration significantly increased the activities of antioxidant enzymes (SOD and CAT) in the serum and enhanced GSH-PX activity in the liver of quail. The results obtained in this experiment and those of previous studies show that PSEs enhance the antioxidant capacity of livestock and poultry. Some of the differences that exist may be due to differences in animal breeds, feeding stages, PSE absorption efficiency, and the amount of additives; e.g., aging can cause an increase in the body’s ROS and protein carbonyl levels [74], which can lead to a higher need for antioxidants.

We analyzed the relationship between egg weight, albumen height, and Haugh unit and antioxidant indexes by Pearson’s correlation, which showed that egg weight and Haugh unit were negatively correlated with NO in serum and MDA and NO content in the ovary. Egg-breaking strength was positively correlated with NO content in serum and MDA and NO content in the ovary. Albumen height was negatively correlated with MDA in the liver, NO in serum, and MDA and NO in the ovary. Therefore, we can assume that PSEs can improve the antioxidant capacity of the animal organism, thereby promoting the health of laying hens and improving egg characteristics.

### 4.4. Analysis of Changes in Metabolic Pathway by Liver Untargeted Metabolomics

In this study, a hepatic metabolomics analysis of laying hens revealed metabolic changes and screened for 12 metabolic pathways that mainly affect Genetic Information Processing and Metabolism in the organism. Among the pathways with significant changes were Aminoacyl-tRNA biosynthesis; Phenylalanine, tyrosine, and tryptophan biosynthesis; Glycine, serine, and threonine metabolism; Lysine degradation; Nucleotide metabolism and ABC transporter metabolism.

Aminoacyl-tRNA is a type of tRNA (also known as transfer RNA) that binds to its corresponding amino acid. Its role is to deliver the amino acid to the ribosome, where it is added to the growing polypeptide chain [75]. The fact that specific amino acids can be added to the corresponding tRNA is very important. This means that the DNA can translate the correct protein and perform the appropriate physiological role [76,77]. In the liver of the experimental groups, the levels of Lysine, L-Tryptophan, Phenylalanine, and L-Tyrosine in the Aminoacyl-tRNA biosynthesis pathway were higher than those of the control group. This implies that the addition of phytosterol esters to the ration has a promoting effect on Aminoacyl-tRNA biosynthesis. Because Aminoacyl-tRNA biosynthesis is closely related to proteins, which are composed of amino acids, we studied amino acid metabolism in the liver and found that L-Tryptophan, Phenylalanine, L-Tyrosine, 5-Aminolevulinic Acid, L-Picolinate, and 5-Hydroxylysine levels were elevated in hepatocytes, whereas creatine levels were decreased. In addition, we used Pearson’s correlation analysis, which showed that the relationship between Haugh unit and albumen height correlated significantly positively with twelve metabolites. TP in the liver, T-AOC, and SOD in plasma correlated significantly positively with the metabolites 5-Hydroxylysine, Ureidopropionic acid, L-Phenylalanine, L-Tryptophan, L-Tyrosine, 5-Aminolevulinic Acid, L-Lysine, and L-Pipecolic acid. Additionally, the TG, TC, and LDL content in the liver, NO and MDA content in the ovary, and NO content in plasma correlated negatively with the above eight amino acid contents. The above findings suggest that the addition of phytosterol esters to the diet promotes Aminoacyl-tRNA biosynthesis and amino acid metabolism, while these two metabolic pathways may also be strongly correlated with increased TP and antioxidant capacity. Therefore, we suggest that PSEs may enhance protein synthesis in laying hens by affecting Aminoacyl-tRNA biosynthesis and other pathways [78], promoting three amino acid metabolic pathways, namely, the Phenylalanine, tyrosine, and tryptophan biosynthesis; Glycine, serine, and threonine metabolism; Lysine degradation, which may promote the animal’s growth, and improve the egg quality and antioxidant capacity of the organism. However, in Nucleotide metabolism, levels of Cytosine, Uracil, and Inosine decrease while Ureidopropionic acid increases in hepatocytes. We speculate that increased amino acid metabolism depletes nucleotides in the liver, leading to lower Cytosine, Uracil, and Inosine levels.

## 5. Conclusions

The study demonstrated that supplementing the diet of Hy-Line Brown laying hens at peak laying stage with phytosterol esters resulted in improved egg characteristics, antioxidant capacity, liver function, and a reduction in symptoms of fatty liver hemorrhagic syndrome. The changes observed in liver metabolism suggest that the mechanism of action may be related to pathways such as Aminoacyl-tRNA biosynthesis and amino acid metabolism. In conclusion, this study has shown that phytosterol esters can be used as safe and effective dietary additives, providing an alternative to antibiotics.

## Figures and Tables

**Table 1:** Composition and nutrient level of the basal diet (air-dry basis).

Ingredients (%)	Content	Nutrient Composition	Content
Corn	61	ME ^2^ (kcal·kg^−1^)	2629.9
Soybean meal	25.5	Crude protein (%)	16.38
Soybean oil	1.5	Calcium (%)	3.14
Limestone	8	Available phosphorus (%)	0.33
Premix ^1^	4	Lysine (%)	1.18
Total	100	Methionine (%)	0.45
		Threonine (%)	0.63
		Cystine (%)	0.27

^1^ Premix provided the following per kilogram of diet: NaCl 3.2 g; VA 8000 IU; VD3 2200 IU; VE 5 IU; VK3 2.5 mg; VB1 2.5 mg; VB2 5.0 mg; VB6 2.0 mg; VB12 0.2 mg; niacin 50 mg; pantothenic acid 6 mg; folic acid 1.0 mg; Cu 5.0 mg; Fe 60 mg; Mn 40 mg; Zn 50 mg. ^2^ Metabolizable energy (ME) based on calculated values; others were analyzed values.

**Table 2:** Effects of phytosterol ester supplementation on performance parameters of laying hens.

Items	Phytosterol Esters/(mg/kg)				SEM	*p*-Value
	0	10	20	40		
Feed intake (g/d/hen)	119.37	115.39	119.39	121.20	0.79	0.052
Laying rate (%)	88.47	89.44	89.25	90.51	0.01	0.6
Feed-to-egg ratio (feed/kg egg)	2.16	2.17	2.19	2.16	0.01	0.915
Average egg weight (g)	60.49 ^b^	60.26 ^b^	62.80 ^a^	62.98 ^a^	0.34	<0.001

^a,b^ Means with different superscripts within each row are significantly different (*p* < 0.05). One-way ANOVA was used to analyze the data (means ± SEM).

**Table 3:** Effects of phytosterol ester supplementation on egg quality of laying hens.

Items	Phytosterol Esters/(mg/kg)				SEM	*p*-Value
	0	10	20	40		
Egg breaking strength (kgf)	5.33 ^a^	5.06 ^ab^	4.61 ^c^	4.78 ^bc^	0.09	0.011
Albumen height (mm)	6.32 ^c^	8.65 ^b^	9.59 ^a^	8.51 ^b^	0.29	<0.001
Haugh unit (B)	76.86 ^b^	93.36 ^a^	94.79 ^a^	89.47 ^a^	1.78	<0.001
Yolk color depth	6.87	6.93	7.03	6.97	0.05	0.74
Eggshell thickness (mm)	0.42	0.42	0.43	0.43	0.00	0.169

^a,b,c^ Means with different superscripts within each row are significantly different (*p* < 0.05). One-way ANOVA was used to analyze the data (means ± SEM).

**Table 4:** Effects of phytosterol ester supplementation on eggshell ultrastructure of laying hens.

Items (μm)	Phytosterol Esters/(mg/kg)				SEM	*p*-Value
	0	10	20	40		
Total thickness	3723.24 ^a^	3661.89 ^ab^	3631.99 ^b^	3638.07 ^b^	12.28	0.026
Effective thickness	2801.20 ^a^	2784.18 ^ab^	2684.57 ^bc^	2616.92 ^c^	21.74	0.004
Mammillary thickness	742.62 ^b^	822.04 ^b^	929.54 ^a^	931.85 ^a^	20.68	0.001
Mammillary width	1056.03 ^a^	804.65 ^b^	606.11 ^c^	653.75 ^c^	41.42	<0.001

^a,b,c^ Means with different superscripts within each row are significantly different (*p* < 0.05). One-way ANOVA was used to analyze the data (means ± SEM).

**Table 5:** Effects of phytosterol ester supplementation on amino acids (g/100 g) in egg yolk of laying hens.

Items (μg/g)	Phytosterol Esters/(mg/kg)				SEM	*p*-Value
	0	10	20	40		
Valine	20.84	22.43	22.02	20.72	0.40	0.382
Glycine	5.54	4.79	4.80	5.49	0.19	0.336
Alanine	22.89	22.99	23.05	24.23	0.42	0.695
Serine	28.64	26.37	28.84	29.47	0.84	0.652
Proline	22.79	21.64	21.25	23.50	0.58	0.551
Threonine	12.44	12.33	12.70	12.80	0.09	0.236
Isoleucine	19.69 ^b^	20.47 ^ab^	21.15 ^ab^	22.33 ^a^	0.38	0.01
Leucine	25.65 ^b^	25.85 ^ab^	26.80 ^ab^	26.93 ^a^	0.22	0.029
Asparagine	12.30	12.29	12.37	12.56	0.06	0.336
Aspartic	23.43	23.59	23.87	24.25	0.19	0.472
Homocysteine	11.47	11.43	12.98	12.91	0.42	0.412
Glutamine	24.59	23.01	25.51	25.19	0.51	0.337
Lysine	1.25	1.44	0.61	0.95	0.17	0.370
Glutamic	53.95	53.79	54.69	55.92	0.49	0.455
Methionine	12.79	13.32	14.60	14.39	0.34	0.186
Histidine	5.37	5.81	6.2	4.28	0.44	0.466
Phenylalanine	12.31 ^b^	13.41 ^ab^	13.70 ^ab^	14.90 ^a^	0.43	0.047
Arginine	18.95	19.17	17.75	16.95	0.42	0.214
Tyrosine	7.62	8.97	10.18	8.67	0.74	0.738
Tryptophan	22.66	22.39	23.36	23.61	0.24	0.233

^a,b^ Means with different superscripts within each row are significantly different (*p* < 0.05). One-way ANOVA was used to analyze the data (means ± SEM).

**Table 6:** Effects of phytosterol ester supplementation on amino acid metabolite in liver of laying hens.

Items (ng/g)	Phytosterol Esters/(mg/kg)				SEM	*p*-Value
	0	10	20	40		
L-Phenylalanine	852.00 ^b^	1236.67 ^a^	1208.57 ^a^	1193.57 ^a^	32.07	<0.001
Leucyl-Lysine	77.48 ^b^	196.00 ^a^	168.27 ^a^	152.86 ^ab^	11.06	0.008
Glutamyl-Leucyl-Arginine	19.59 ^b^	48.33 ^a^	47.83 ^a^	41.29 ^ab^	3.48	0.046
Aspartyl-Isoleucine	63.12 ^b^	112.82 ^a^	102.66 ^ab^	101.90 ^ab^	5.62	0.044
L-Tryptophan	548.20 ^b^	781.33 ^a^	726.57 ^a^	687.93 ^ab^	22.75	0.014
L-Tyrosine	599.40 ^b^	736.00 ^a^	706.86 ^ab^	741.36 ^a^	17.38	0.033

^a,b^ Means with different superscripts within each row are significantly different (*p* < 0.05). One-way ANOVA was used to analyze the data (means ± SEM).

**Table 7:** Effects of phytosterol ester supplementation on biochemical indexes of liver.

Items ^1^	Phytosterol Esters/(mg/kg)				SEM	*p*-Value
	0	10	20	40		
TP (g/gprot)	13.84 ^c^	15.55 ^bc^	18.89 ^a^	16.86 ^ab^	0.54	0.002
ALB (g/gprot)	4.46	3.21	2.95	2.54	0.30	0.116
AKP (IU/gprot)	206.65 ^a^	190.63 ^b^	180.95 ^c^	175.05 ^c^	2.83	<0.001
LDL-C (mmol/gprot)	0.08 ^a^	0.07 ^a^	0.04 ^b^	0.03 ^c^	0.00	<0.001
HDL-C (mmol/gprot)	3.26	4.02	4.26	6.25	0.41	0.052
TC (mmol/gprot)	17.80 ^a^	14.26 ^a^	6.28 ^b^	3.73 ^b^	1.43	<0.001
TG (mmol/gprot)	11.45 ^a^	3.33 ^b^	2.38 ^b^	2.89 ^b^	0.81	<0.001

^a,b,c^ Means with different superscripts within each row are significantly different (*p* < 0.05). One-way ANOVA was used to analyze the data (means ± SEM). ^1^ TP: total protein; ALB: albumin; AKP: alkaline phosphatase; LDL-C: low-density lipoprotein cholesterol; HDL-C: high-density lipoprotein cholesterol; TC: total cholesterol; TG: triglycerides.

**Table 8:** Effects of phytosterol ester supplementation on antioxidant capacity in laying hens.

Items ^1^	Phytosterol Esters/(mg/kg)				SEM	*p*-Value
	0	10	20	40		
Liver						
CAT (U/mgprot)	67.46	70.30	71.06	69.61	0.69	0.303
SOD (U/mgprot)	153.20	154.54	146.54	151.98	2.65	0.752
T-AOC (U/mgprot)	4.35	4.80	4.99	5.13	0.12	0.081
MDA (nmol/mgprot)	0.53 ^b^	0.47 ^b^	0.34 ^ab^	0.37 ^a^	0.03	0.044
NO (umol/gprot)	1.58	1.05	0.87	0.82	0.13	0.141
Ovary						
T-AOC (U/mgprot)	3.73	4.04	2.94	2.94	0.25	0.300
MDA (nmol/mgprot)	2.61 ^d^	2.16 ^c^	1.33 ^b^	0.94 ^a^	0.15	<0.001
NO (umol/gprot)	8.35 ^a^	4.54 ^bc^	5.07 ^b^	3.95 ^c^	0.39	<0.001
Plasma						
SOD (U/mL)	22.95 ^b^	23.17 ^a^	23.20 ^a^	23.32 ^a^	0.04	0.009
T-AOC (U/mL)	3.67 ^b^	4.50 ^a^	4.08 ^ab^	4.48 ^a^	0.10	0.001
MDA (nmol/mL)	1.28	1.12	1.17	0.82	0.07	0.090
NO (umol/L)	9.53 ^a^	7.49 ^b^	7.38 ^b^	7.15 ^b^	0.29	0.004

^a,b,c,d^ Means with different superscripts within each row are significantly different (*p* < 0.05). One-way ANOVA was used to analyze the data (means ± SEM). ^1^ prot, protein; CAT, catalase; SOD, total superoxide dismutase; T-AOC, total antioxidant capacity; MDA, malondialdehyde; NO, nitric oxide.

**Table 9:** KEGG pathway enrichment analysis of 84 differential metabolites (*p* < 0.05).

Num	First Category	Second Category	Pathway Description	Ratio ^1^	*p*-Value
1	Genetic Information Processing	Translation	Aminoacyl-tRNA biosynthesis	4/23	<0.001
2	Metabolism	Amino acid metabolism	Phenylalanine, tyrosine, and tryptophan biosynthesis	3/23	<0.001
3	Metabolism	Amino acid metabolism	Glycine, serine, and threonine metabolism	3/23	0.002
4	Metabolism	Amino acid metabolism	Lysine degradation	3/23	0.002
5	Metabolism	Global and overview maps	Nucleotide metabolism	3/23	0.004
6	Metabolism	Nucleotide metabolism	Pyrimidine metabolism	3/23	0.005
7	Environmental Information Processing	Membrane transport	ABC transporters	4/23	0.006
8	Metabolism	Metabolism of cofactors and vitamins	Pantothenate and CoA biosynthesis	2/23	0.011
9	Metabolism	Metabolism of other amino acids	beta-Alanine metabolism	2/23	0.012
10	Metabolism	Amino acid metabolism	Phenylalanine metabolism	2/23	0.029
11	Organismal Systems	Endocrine system	Melanogenesis	1/23	0.032
12	Metabolism	Metabolism of other amino acids	D-Amino acid metabolism	2/23	0.049

^1^ Ratio: The proportion of metabolites in the target metabolite set annotated to this pathway versus the total number of metabolites in the set (whose metabolites can be annotated to the KEGG pathway).

**Table 10:** Correlations between egg quality and antioxidant capacity ^1^.

Items	Liver	Plasma			Ovary	
	MDA	SOD	T-AOC	NO	MDA	NO
Egg weight	−0.302	0.368	0.296	−0.465 *	−0.525 **	−0.560 **
Egg breaking strength	0.381	−0.188	−0.403	0.459 *	0.546 **	0.444 *
Albumen height	−0.423 *	0.388	0.297	−0.632 **	−0.584 **	−0.648 **
Haugh unit	−0.265	0.347	0.404	−0.634 **	−0.529 **	−0.677 **

* The statistical difference (*p* < 0.05). ** Highly significant difference (*p* < 0.01). ^1^ T-AOC, total antioxidant capacity; SOD, total superoxide dismutase; MDA, malondialdehyde; NO, nitric oxide.

**Table 11:** Correlations between egg characteristics and biochemical indexes of liver ^1^.

Items	Liver			
	TP	TC	TG	LDL
Egg weight	0.468 *	−0.380	−0.708 **	−0.525 **
Egg breaking strength	−0.475 *	0.525 **	0.542 **	0.585 **
Albumen height	0.641 **	−0.478 *	−0.784 **	−0.538 **
Haugh unit	0.619 **	−0.331	−0.793 **	−0.362

* The statistical difference (*p* < 0.05). ** Highly significant difference (*p* < 0.01). ^1^ TP: total protein; TC: total cholesterol; TG: triglycerides; LDLC: low-density lipoprotein cholesterol.

## Data Availability

The data are contained within the article. The unprocessed metabolomics information is stored in the MetaboLights public archive, accessible under the code MTBLS9736.

## References

[1] Miao Y.F., Gao X.N., Xu D.N., Li M.C., Gao Z.S., Tang Z.H., Mhlambi N.H., Wang W.J., Fan W.T., Shi X.Z. (2021). Protective Effect of the New Prepared Atractylodes Macrocephala Koidz Polysaccharide on Fatty Liver Hemorrhagic Syndrome in Laying Hens. Poult. Sci..

[2] Shini A., Shini S., Bryden W.L. (2019). Fatty Liver Haemorrhagic Syndrome Occurrence in Laying Hens: Impact of Production System. Avian Pathol..

[3] Choi Y.I., Ahn H.J., Lee B.K., Oh S.T., An B.K., Kang C.W. (2012). Nutritional and Hormonal Induction of Fatty Liver Syndrome and Effects of Dietary Lipotropic Factors in Egg-Type Male Chicks. Asian-Australas. J. Anim. Sci..

[4] Yang F., Ruan J.M., Wang T.C., Luo J.R., Cao H.B., Song Y.L., Huang J.Z., Hu G.L. (2017). Improving Effect of Dietary Soybean Phospholipids Supplement on Hepatic and Serum Indexes Relevant to Fatty Liver Hemorrhagic Syndrome in Laying Hens. Anim. Sci. J..

[5] Duan Q.B., Yu P.T. (2019). Causes and control measures of sudden death syndrome in broilers. Modern Rural Sci. Technol..

[6] Ding J.X., He S.J., Liu D.Y., Li J. (2018). The effect of diet supplemented with selenium-enriched yeast on production performance of Hy-Line Brown layers in summer season. Heilongjiang J. Anim. Sci. Vet. Med..

[7] Wang J., Qian X., Gao Q., Lv C.M., Xu J., Jin H.B., Zhu H. (2018). Quercetin Increases the Antioxidant Capacity of the Ovary in Menopausal Rats and in Ovarian Granulosa Cell Culture In Vitro. J. Ovarian Res..

[8] Peters A.E., Mihalas B.P., Bromfield E.G., Roman S.D., Nixon B., Sutherland J.M. (2020). Autophagy in Female Fertility: A Role in Oxidative Stress and Aging. Antioxid. Redox Sign..

[9] Deng T., Huang Y., He B.Q., Li J.X., Cao Y.P. (2014). Investigation progress of preparation methods of phytosterol esters. Cereals Oils.

[10] Cheng Q.W., Meng L.L., Feng L., Xu J.R., Teng Z.M. (2016). Study on the Preparation of Phytosterols Stearic. Food Res. Dev..

[11] Xu Q.Q., Jin W.B., Su B.G., Yang Y.W., Ren Q.L. (2014). Progress in the Chemical Synthesis, Separation and Purification of Phytosterol Esters. J. Chin. Cereals Oils Assoc..

[12] Zhao Y., Tang G.S., Hou Y.Y., Niu S.J., Gao Y.G., Han X., Zhang Y.Y., Shen Y.L., Zhang L.X. (2015). Research on synthesis technology of phytosterol esters. J. Food Saf. Qual..

[13] Qing Y.Q. (2023). Effects of Dietary Supplement with Phy-Sterol Esters on Production Performance, Egg Quality and Serum Biochemical Indexes of Quail. Master’s Thesis.

[14] Peng J., Chen L.P., Bei Y.J., Ding X.Y., Zhou F. (2021). Physiological functions and application in animal production of phytosterols. Feed Res..

[15] Pollak O.J. (1953). Reduction of Blood Cholesterol in Man. Circulation.

[16] Zhao W.J. (2019). Effects of Maternal Dietary Supplementation of Phytosterol Esters in Mammalian during Gestation on Muscle Development of Its Offspring. Master’s Thesis.

[17] Fernandes P., Cabral J.M.S. (2007). Phytosterols: Applications and Recovery Methods. Bioresour. Technol..

[18] Uddin M.S., Ferdosh S., Akanda M.J.H., Ghafoor K., Rukshana A.H., Ali M.E., Yunus K., Fauzi M.B., Hadijah S., Shaarani S.M. (2018). Techniques for the Extraction of Phytosterols and Their Benefits in Human Health: A Review. Sep. Sci. Technol..

[19] Zhang X., Chen S.M., Wu N., Wang T., Pei X.W., Jiang L.Z., Han C.P., Yu D.Y. (2019). Selective Modification of MCM-41 Immobilized Lipase and Its Application in Sterol Ester Synthesis. Food Sci..

[20] Guo Y. (2021). Plant Sterol Ester of Alpha-Linolenic Improved Nonalcoholic Fatty Liver Disease by Inhibiting Endoplasmic Reticulum Stress. Master’s Thesis.

[21] Li X.Y., Zheng M.M., Guo Y., Wang L.Q., Xue T.L., Han H. (2020). Protective of Plant Sterol Ester of α-Linolenic Acid from Non-Alcojolice Fatty Liver DiseaseI by Inhibiting Oxidative Stress. Acta Nutr. Sin..

[22] Wang L.Q., Zheng M.M., Xue T.L., Li J., Pei L.Y., Han H. (2021). Plant Sterol Ester of α-Linolenic Acid Improves Liver Fibrosis by Regulating TGF-β1/Smad Signaling Psthway in Mice. Acta Nutr. Sin..

[23] Zhang X.F., Xue Y.T., Zhang D.J. (2020). Advances in Research on Phytosterols Protecting Gastric Mucosa and Anti-Gastrointestinal Tumors. Genom. Appl. Biol..

[24] Xue Y.T., Zhang X.F., Zhang D.J. (2019). Research progress of the blood lipid lowering effect of phytosterol. West China J. Pharm. Sci..

[25] Wang X.K., Chen W., Zhang T.J., Yi Z.G., Gu Y.L., Li T. (2023). Research and application prospect of phytosterols(esters). China Surfactant Deterg. Cosmet..

[26] Zhang N., Yang K.L., Zhang S., Hao J.Y., Deng S.T., Yang W.G., Liu S.P., Sun J.L., Fang R.J. (2023). Effects of Phytosterol Ester on Laying Performance, Egg Quality, Liver Antioxidant Capacity and Yolk Precursor Synthesis of Laying Hens during Late Laying Period. Chin. J. Anim. Nutr..

[27] Ding X.Q., Yuan C.C., Huang Y.B., Jiang L., Qian L.C. (2021). Effects of Phytosterol Supplementation on Growth Performance, Serum Lipid, Proinflammatory Cytokines, Intestinal Morphology, and Meat Quality of White Feather Broilers. Poult. Sci..

[28] Cao C.Y., Sun X.J., Hu Z.Y., Xiao R. (2016). Comparison of the anaesthetic effects of three anaesthetic methods on Leghorn chickens. Lab. Anim. Comp. Med..

[29] Fathi M.M., El-Dlebshany A.E., El-Deen M.B., Radwan L.M., Rayan G.N. (2016). Effect of Long-Term Selection for Egg Production on Eggshell Quality of Japanese Quail (*Coturnix japonica*). Poult. Sci..

[30] Bain M.M. (1992). Eggshell Strength: A Relationship between the Mechanism of Failure and the Ultrastructural Organization of the Mammillary Layer. Br. Poult. Sci..

[31] Solomon S.E. (1997). Structural Variations in the Mammillary Layer. Egg & Eggshell Quality.

[32] Li C., Al-Dalali S., Zhou H., Xu B. (2022). Influence of Curing on the Metabolite Profile of Water-Boiled Salted Duck. Food Chem..

[33] Li Z., Wu Y., Hu J., Yang G., Wang Z., Sun J. (2022). Dissection of the Response Mechanism of Alfalfa under Phosphite Stress Based on Metabolomic and Transcriptomic Data. Plant Physiol. Biochem..

[34] Kong X., Guo Z., Yao Y., Xia L., Liu R., Song H., Zhang S. (2022). Acetic Acid Alters Rhizosphere Microbes and Metabolic Composition to Improve Willows Drought Resistance. Sci. Total Environ..

[35] Yuan C.C., Fan J.H., Jiang L., Ye W.X., Chen Z., Wu W.Z., Huang Q.X., Qian L.C. (2023). Integrated Analysis of Gut Microbiome and Liver Metabolome to Evaluate the Effects of Fecal Microbiota Transplantation on Lipopolysaccharide/D-Galactosamine-Induced Acute Liver Injury in Mice. Nutrients.

[36] Hu Q.L., Huang D., Ping F.J. (2014). Physiological functions of phytosterols and their research and application in animal production. Feed Res..

[37] Zhou B.L. (1992). Applications of phytosterols. China Oils Fats.

[38] Qian L.C., Jiang L., Ye W.X., Yu D.Y., Wang Z.G., Han X.Y., Zhao P.J. (2022). A Method of Preparing Phytosterol Esters for Feeding, Phytosterol Esters and Their Applications.

[39] Yuan C. (2016). Research on the Mechanism of L-Arginine on the Regulation Offeed Intake and Tissue Protein Metabolism in Laying Hens. Ph.D. Thesis.

[40] Zhu Y.X. (2020). Key technology of forest ecological farming for Hyland Brown laying hens. Livest. Vet. Sci. Technol. Inform..

[41] Zhou C.J. (2014). Biosafety Evaluation of Phytosterol as Feed Additivein Laying Hens. Master’s Thesis.

[42] Zhou C.J., Shi S.R., Tong H.B., Zou J.M., Wang Z.Y. (2013). Phytosterol: Effects on Production Performance, Blood Routine Parameters and Serum Biochemical Parameters of Laying Hens. Chin. J. Anim. Nutr..

[43] Wang L.C., Gu W.T., Zhou Y.M., Wan T. (2008). Effects of Phytosterols on Performance, Cholesterol Content in Egg Yolk and Reproductive Hormones in Serum of Laying Hens in Late Period of Laying. J. Chin. Cereals Oils Assoc..

[44] Gu W.T. (2007). Application of Phytosterols and Its Mechanisms in Regulation of Cholesterol Metabolism in Meat-Strain Ducks. Master’s Thesis.

[45] Chang L.L., Shen Y.R., Zhou C.J., Wang Z.Y., Tong H.B., Zou J.M., Shi S.R. (2014). Effects of High-Dose Phytosterol on Performance, Egg Quality and Lipid Metabolism of Laying Hens. China J. Anim. Nutr..

[46] Yuan C., Song H.H., Zhang X.Y., Jiang Y.J., Zhang A.T., Azzam M.M., Zou X.T. (2014). Effect of Expanded Cottonseed Meal on Laying Performance, Egg Quality, Concentrations of Free Gossypol in Tissue, Serum and Egg of Laying Hens. Anim. Sci. J..

[47] Zhang Y.N. (2018). Dietary Manganese Supplementation Modulated Eggshell Quality in Laying Hens. Ph.D. Thesis.

[48] Zhang Y.N., Zhang H.J., Wu S.G., Wang J., Qi G.H. (2018). Dietary Manganese Supplementation Affects Mammillary Knobs of Eggshell Ultrastructure in Laying Hens. Poult. Sci..

[49] Ketta M., Tůmová E. (2016). Eggshell Structure, Measurements, and Quality-Affecting Factors in Laying Hens: A Review. Czech J. Anim. Sci..

[50] Athanasiadou D., Jiang W., Goldbaum D., Saleem A., Basu K., Pacella M.S., Böhm C.F., Chromik R.R., Hincke M.T., Rodríguez-Navarro A.B. (2018). Nanostructure, Osteopontin, and Mechanical Properties of Calcitic Avian Eggshell. Sci. Adv..

[51] Wen G.L. (2019). Regulation Mechanism of Glucosamine on Eggshell Quality in Laying Hens. Master’s Thesis.

[52] Xiao J.F. (2014). Effect of Dietary Manganese Sources and Supplemental Levels on Eggshell Quality of Laying Hens. Ph.D. Thesis.

[53] Ma Y.X., Wu Y.X., Shao R., Miao L. (2022). Stigmasterol inhibits the contraction of rat prostatic stromal cells by inhibiting calcium influx. Tianjin J. Tradit. Chin. Med..

[54] Zhao Z.B. (2019). Cholesterol Attenuated HCC Progression through Modulating IP3Rs Mediated Calcium Release. Master’s Thesis.

[55] Wang G. (2022). Cholesterol Mediates by the Inhibitory Effect of Shear Force on Calcium-Activated Chloride Channel TMEM16A Current. Master’s Thesis.

[56] Zhou H.Y. (2017). The Mechanisms of Phytosterol Ester on Non-Alcoholic Fatty Liver Disease Metabolism. Master’s Thesis.

[57] Chen Z.F., Gao F., Xu H.B. (2008). Advances in the mechanism of phytosterols affecting cholesterol metabolism. J. Environ. Hyg..

[58] Liu X., Pan Y., Shen Y., Liu H., Zhao X., Li J., Ma N. (2022). Protective Effects of Abrus Cantoniensis Hance on the Fatty Liver Hemorrhagic Syndrome in Laying Hens Based on Liver Metabolomics and Gut Microbiota. Front. Vet. Sci..

[59] De Smet E., Mensink R.P., Plat J. (2012). Effects of Plant Sterols and Stanols on Intestinal Cholesterol Metabolism: Suggested Mechanisms from Past to Present: Molecular Nutrition & Food Research. Mol. Nutr. Food Res..

[60] Plat J., Baumgartner S., Mensink R.P. (2015). Mechanisms Underlying the Health Benefits of Plant Sterol and Stanol Ester Consumption. J. AOAC Int..

[61] Wen C., Wu P., Yang W., Zhou Y. (2012). Effect of Different Phytosterols on Lipid Metabolism of Laying Hens. J. Chin. Cereals Oils Assoc..

[62] Jia D.H., Zhou Y.M., Wang T. (2007). Effects of Phytosterol on Cholesterol and Protein Level and Antioxidation Enzyme Activity in Serum of Broilers. J. Chin. Cereals Oils Assoc..

[63] Sun L. (2014). A Primary Research of The Application of Phytostreols in Livestock Production. Master’s Thesis.

[64] Liu W.W. (2007). Study on Blood-Fat-Lowering Effects of Phytosterol Esters in Hyperlipidemia Rats. Master’s Thesis.

[65] Wang Z.Y. (2021). Effects of 25-(OH)D3, Vitamin C, 4,7-dihydroxyisoflavoneon Chicken eggshell Quality and Calcium Metabolism. Master’s Thesis.

[66] Newmeyer D.D., Ferguson-Miller S. (2003). Mitochondria: Releasing Power for Life and Unleashing the Machineries of Death. Cell.

[67] Panda A.K., Rao S.S.R., Raju M.V., Sharma S.S. (2008). Effect of Probiotic (*Lactobacillus sporogenes*) Feeding on Egg Productionand Quality, Yolk Cholesterol and Humoralimmune Response of White Leghorn Layerbreeders. J. Sci. Food Agric..

[68] Surai P.F., Sparks N.H.C. (2000). Tissue-Specific Fatty Acid and *α*-Tocopherol Profiles in Male Chickens Depending on Dietary Tuna Oil and Vitamin E Provision. Poult. Sci..

[69] Reynard M., Savory C.J. (1999). Stress-Induced Oviposition Delays in Laying Hens: Duration and Consequences for Eggshell Quality. Brit. Poult. Sci..

[70] Gong H., Sun F.G., Huang Z.Y., Pan Z.C., Li Z.H., Zou S.L. (2020). Effects of phytosterol on growth performance, physical indicators, muscle composition and hepatic biochemical index of Micropterus salmoides. J. Aquacult..

[71] Yuan C., Ding X., Jiang L., Ye W., Xu J., Qian L. (2021). Effects of Dietary Phytosterols Supplementation on Serum Parameters, Nutrient Digestibility and Digestive Enzyme of White Feather Broilers. Ital. J. Anim. Sci..

[72] Huang Z.Y., Li Z.H., Gong H., Sun F.G., Zou S.L. (2019). Effects of Phytosterol on Growth Performance and Serum Biochemical indexes of Qingyuan Partridge Chicken. Guangdong Feed.

[73] Li Z.H., Huang Z.Y., Pan Z.C., Sun F.G., Zhao H.H. (2019). Effects of Phytosterol on Growth Performance, Serum Lipid Metabolism Indicators and Hepatopancreas Antioxidant Indicators of Tilapia (*Oreochromis niloticus*). Chin. J. Anim. Nutr..

[74] Luceri C., Bigagli E., Femia A.P., Caderni G., Giovannelli L., Lodovici M. (2018). Aging Related Changes in Circulating Reactive Oxygen Species (ROS) and Protein Carbonyls Are Indicative of Liver Oxidative Injury. Toxicol. Rep..

[75] Bullwinkle T.J., Ibba M., Kim S. (2014). Emergence and Evolution. Aminoacyl-tRNA Synthetases in Biology and Medicine.

[76] Gomez M.A.R., Ibba M. (2020). Aminoacyl-tRNA Synthetases. RNA.

[77] Ibba M., Söll D. (2004). Aminoacyl-tRNAs: Setting the Limits of the Genetic Code. Genes Dev..

[78] Takénaka A., Moras D. (2020). Correlation between Equi-Partition of Aminoacyl-tRNA Synthetases and Amino-Acid Biosynthesis Pathways. Nucleic Acids Res..

